# Holt–Oram Syndrome With Complex Cardiac and Limb Anomalies in an Ethiopian Patient: A Case Report

**DOI:** 10.1155/cric/5488112

**Published:** 2026-08-31

**Authors:** Yitagesu Getachew, Abera Birhanu, Samson Mulugeta, Balew Arega, Begashaw Belay, Amdemeskel Mersha, Banchiaymolu Damtie, Abel Yirga, Eba Fufa, Ibrahim Kemal, Brook Fanuel

**Affiliations:** ^1^ Department of Internal Medicine, Yekatit 12 Hospital Medical College, Addis Ababa, Ethiopia

**Keywords:** HOS, limb anomalies, ventricular septal defect

## Abstract

**Introduction:**

Holt–Oram syndrome (HOS) is a rare autosomal dominant disorder characterized by upper limb and congenital heart anomalies, with an estimated incidence of 1 in 100,000 live births.

**Case Report:**

An 18‐year‐old Ethiopian male with a history of congenital heart disease presented with progressive dyspnea and limb deformities. Imaging confirmed a membranous ventricular septal defect, severe pulmonary hypertension, mitral and tricuspid regurgitation, and skeletal anomalies, including thumb hypoplasia and absent distal radial bone.

**Conclusion:**

This case underscores the importance of early diagnosis and multidisciplinary management of HOS, especially in resource‐limited settings where genetic testing is unavailable. Increased awareness can facilitate timely intervention and improve patient outcomes.

## 1. Introduction

Holt–Oram syndrome (HOS), or heart–hand syndrome, represents a rare genetic disorder with an estimated occurrence of 1 in 100,000 live births [1]. It is caused by mutations in the TBX5 gene, located on chromosome 12q24, which plays a crucial role in cardiac and upper limb morphogenesis. It follows an autosomal dominant inheritance pattern with variable expressivity [2, 3].

The diagnosis is not difficult in the presence of typical clinical features and a positive family history. The diagnosis is based on established clinical criteria and can be confirmed by molecular genetic testing in a proportion of cases [4]. The clinical diagnostic criteria of HOS include skeletal and cardiac anomalies or a family history of similar illness. The upper‐limb anomalies are always present [5]. Upper limb anomalies include triphalangeal or absent thumbs, carpal fusion, and hypoplasia of the radius or ulna [6]. Cardiac defects range from atrial and ventricular septal defects (VSDs) to conduction abnormalities and valvular defects [7, 8, 5].

Previously, HOS cases were reported in a registry‐based study in Europe [4] and from a population‐based study in Hungary covering the 1975–1988 period [1]. The findings showed that the clinical presentation and organ involvement were heterogeneous. Despite its characteristic clinical features, HOS is underdiagnosed in Ethiopia due to limited access to genetic testing and a lack of awareness among clinicians. Here, we report a case of HOS with a membranous VSD, several pulmonary hypertensions, and multiple congenital anomalies of the upper extremities.

## 2. Case Report

An 18‐year‐old Ethiopian male with a known history of congenital heart disease since childhood has been on the surgical waiting list for corrective cardiac intervention. He has been on oral diuretics (furosemide 40 mg daily, a loop diuretic prescribed to manage volume overload resulting from the congenital heart defect). By inhibiting the Na‐K‐2Cl cotransporter in the thick ascending limb of the loop of Henle, furosemide promotes diuresis, reduces preload, and alleviates symptoms of pulmonary congestion. Currently, he presents with shortness of breath with minimal exercise and orthopnea of two pillows, but there is no paroxysmal nocturnal dyspnea (PND) or body swelling. The clinical significance of orthopnea without PND suggests early to moderate left‐sided heart failure. Orthopnea results from redistribution of fluid from the lower extremities and splanchnic circulation to the central venous and pulmonary circulations upon recumbency, increasing left ventricular filling pressure and causing pulmonary congestion. The absence of PND suggests that the patient′s heart failure has not progressed to more advanced stages or that nocturnal fluid redistribution is insufficient to trigger this symptom. The two‐pillow orthopnea indicates functional Class II–III heart failure, consistent with the echocardiographic findings. Previously, he had a history of repeated hospital admissions for the same complaints. This history of repeated admissions suggests a pattern of clinical decompensation despite medical therapy, indicating disease progression. In the context of uncorrected congenital heart disease, this pattern typically reflects gradual worsening of hemodynamics, with episodes of acute pulmonary congestion triggered by intercurrent infections, arrhythmias, or medication nonadherence. He has no family history of the same illness.

**Figure 1:**
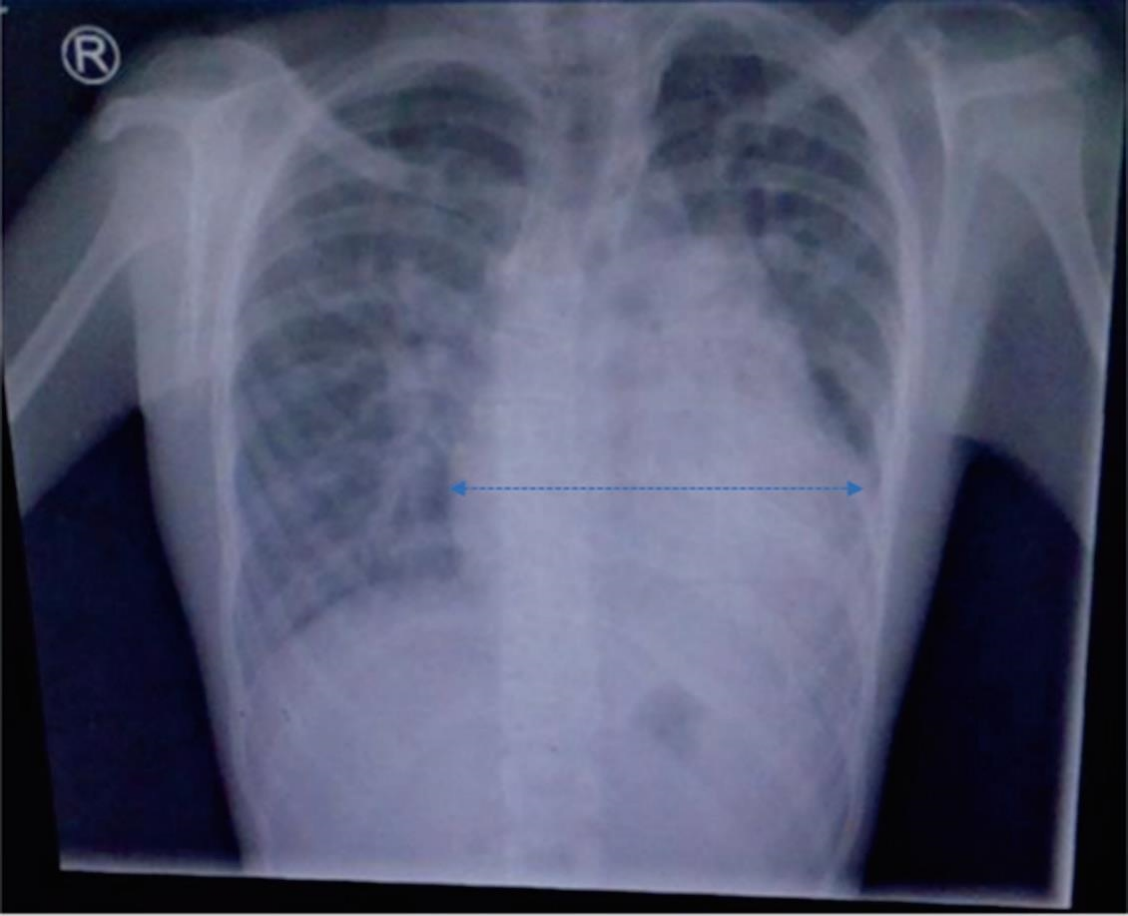
Posteroanterior chest radiograph showing cardiomegaly and Grade I pulmonary edema.

Physical examinations showed an acute, sick‐looking appearance on a chronic basis. The blood pressure was 130/70 mmHg, the respiratory rate was 18 breaths per minute, the heart rate was 90 beats per minute, and he maintained the saturation at atmospheric pressure. Vital signs were measured using a standard mercury sphygmomanometer, physical examination, and pulse oximetry. The chest examination was unremarkable. Chest radiography showed cardiomegaly with evidence of Grade I pulmonary edema (Figure 1).

**Figure 2:**
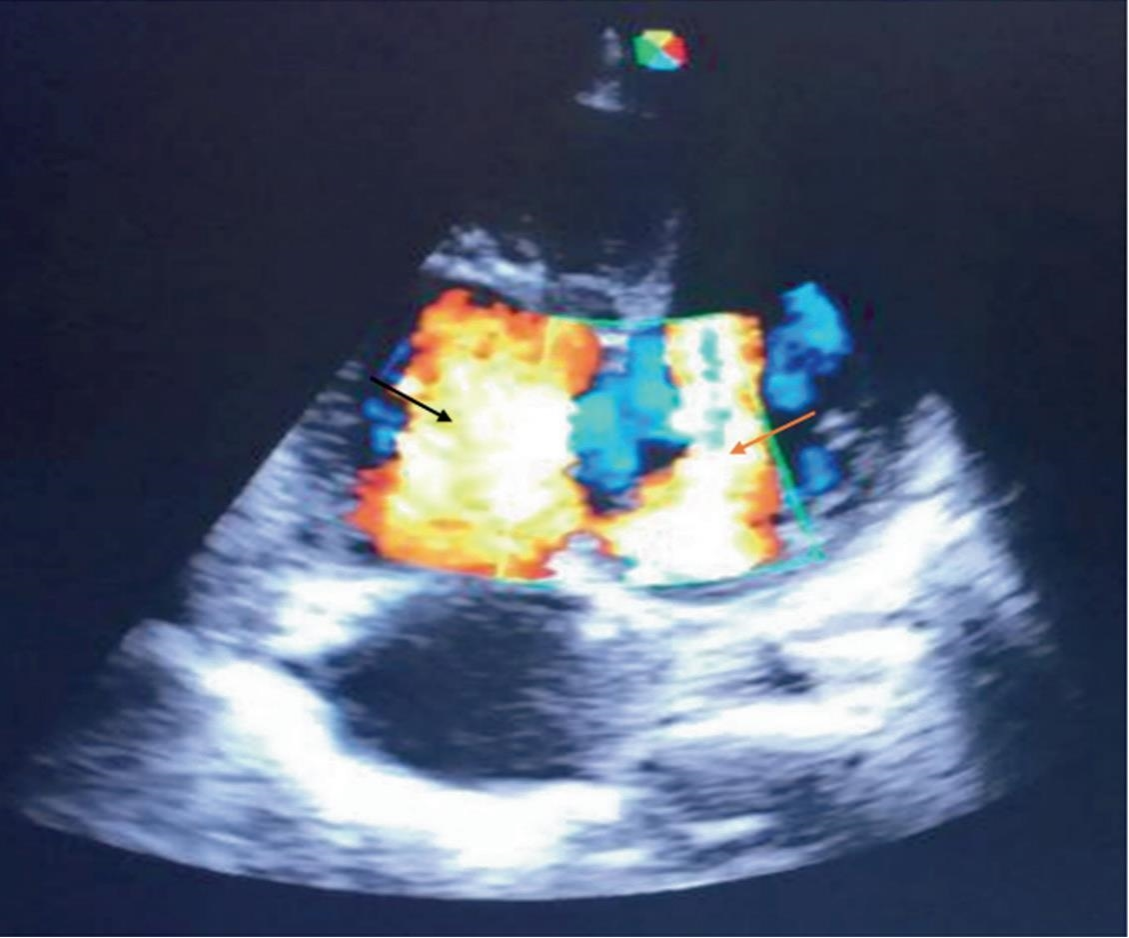
Two‐dimensional echocardiography showing membranous VSD with moderate to severe mitral regurgitation, mild mitral valve prolapse, severe tricuspid regurgitation, and severe pulmonary hypertension.

Precordial examination revealed a bulged precordium, a loud P2 component of the second heart sound, and holosystolic murmurs consistent with mitral and tricuspid regurgitation. Two‐dimensional echocardiography, performed using a commercially available ultrasound system with a 2.5–5 MHz phased‐array transducer following standard American Society of Echocardiography guidelines, confirmed a membranous VSD, severe pulmonary hypertension (RVSP > 50 mmHg), moderate‐to‐severe mitral regurgitation, mild mitral valve prolapses, and severe tricuspid regurgitation (Figure 2). Electrocardiography (ECG) showed a normal sinus rhythm without evidence of conduction abnormalities, though patients with HOS commonly develop atrioventricular (AV) block.

**Figure 3:**
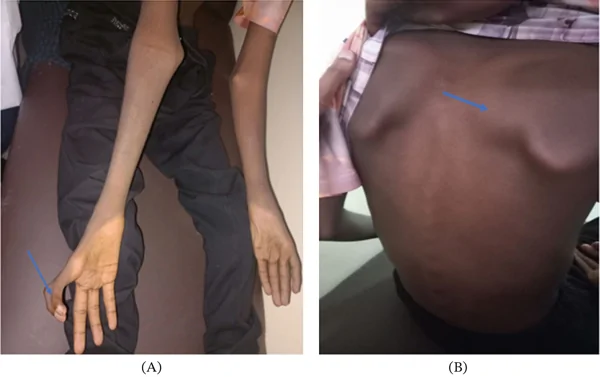
A clinical photograph of the patient showing the (A) fused right thumb and index finger and (B) deformed right scapula.

On abdominal examination, there was no hepatomegaly or splenomegaly, no signs of fluid collection, and no peripheral edema. Musculoskeletal examination revealed fused index and thumb fingers on the right side and an absent thumb on the left side (Figure 3A). The right scapula also has a deformity (Figure 3B).

**Figure 4:**
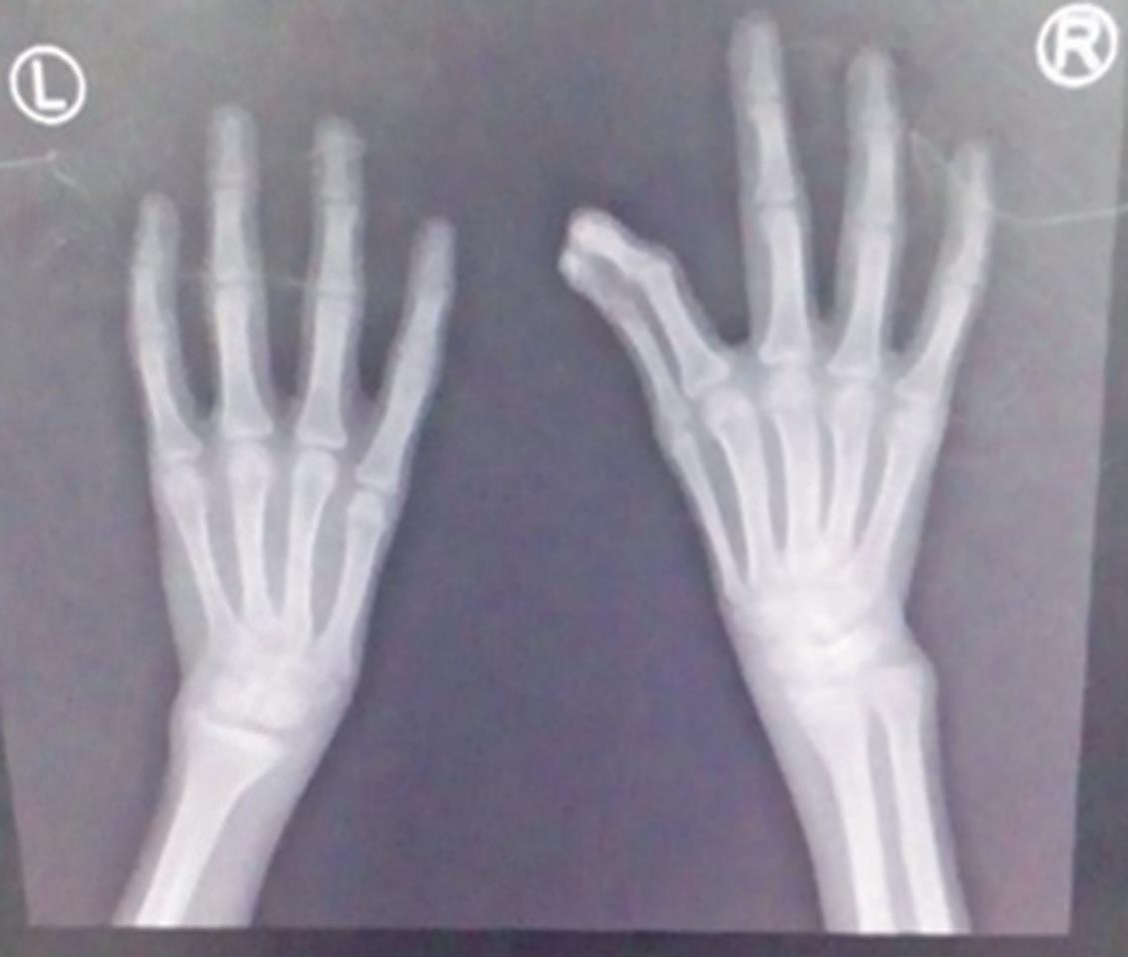
Skeletal radiograph of the upper limb showing absent left distal radial bone and absent first metacarpal and phalanges on the left side, with partially fused left carpal bones, hypoplastic right first digit with soft tissue syndactyly of the first and second digits.

Forearm radiography, obtained using standard digital radiography techniques, showed absent left first digit metacarpal and phalanges, absent distal radial bone, partially fused left carpal bones, as well as hypoplastic right first digit with soft tissue syndactyly of the first and second digits (Figure 4).

The complete blood cell analysis showed no cytopenia or leukocytosis and a normal hemoglobin level for sex (hemoglobin: 14.2 g/dL; white blood cell count: 6.8 × 10^3^/*μ*L; platelet count: 245 × 10^3^/*μ*L). The renal function test (serum creatinine: 0.9 mg/dL; blood urea nitrogen: 14 mg/dL), liver function test (AST: 22 U/L; ALT: 18 U/L; total bilirubin: 0.6 mg/dL), and serum electrolytes (sodium: 138 mEq/L; potassium: 4.1 mEq/L) were also normal. These laboratory investigations were analyzed using standard automated laboratory techniques. Genetic analysis was not affordable or available.

Combining the clinical and imaging findings, we diagnosed the HOS with membranous VSD, several pulmonary hypertensions, and upper extremity anomalies, including the fusing of carpal bones, absent and hypoplastic, and absent distal radial bone. Since the patient had follow‐ups at other centers where cardiac surgery services were provided, we adjusted the diuretics and sent him for his regular follow‐up.

## 3. Discussion

Cardiac anomalies in HOS are highly variable, with the most common defects being atrial septal defects (ASD), particularly ostium secundum, and VSD [8]. Our patient was diagnosed with a membranous VSD, which is consistent with the cardiac spectrum of HOS. However, he also exhibited severe pulmonary hypertension, moderate‐to‐severe mitral regurgitation, mild mitral valve prolapses, and severe tricuspid regurgitation—findings that are less commonly reported in HOS cases [5]. Although ASD and VSD are frequently associated with left‐to‐right shunting, progression to pulmonary hypertension is relatively rare unless there is a large uncorrected defect with Eisenmenger physiology [9]. Another feature of HOS is cardiac conduction abnormalities, which can range from first‐degree AV block to complete heart block [10]. Although our patient did not exhibit conduction delays on the initial ECG, long‐term monitoring is necessary given the progressive nature of these abnormalities. In addition to these straightforward defects, rare and complex cardiac anomalies may also be observed in HOS, including pulmonary stenosis, mitral valve prolapse, as noted in our patient, and various arrhythmias such as AV blocks [11].

The current presentation with progressive dyspnea, orthopnea, and exercise intolerance is directly attributable to the hemodynamic consequences of the uncorrected congenital cardiac defects. The membranous VSD resulted in chronic left‐to‐right shunting, which over time led to volume overload of the right heart, pulmonary hypertension, and ultimately right ventricular dysfunction. The development of severe pulmonary hypertension (RVSP > 50 mmHg) further exacerbated right heart strain, contributing to secondary tricuspid regurgitation. The mitral regurgitation and prolapse likely reflect left ventricular volume overload and annular dilation from the chronic shunt. These progressive hemodynamic alterations culminated in the patient′s current New York Heart Association functional class symptoms, underscoring the importance of timely surgical intervention in such cases.

The principal features of this syndrome encompass deformities of the upper limbs, which may manifest as absent, underdeveloped, or triphalangeal thumbs, along with malformations of the metacarpals and hypoplastic or absent radii or humeri [12]. Our patients have absent left distal radial bone and absent first metacarpal and phalanges on the left side, with partially fused left carpal bones, hypoplastic right first digit with soft tissue syndactyly of first and second digits. The diagnosis is excluded in the presence of lower limb defects, given that the genetic mutation impacts embryonic development during the fourth and fifth weeks of gestation, a period when lower limb differentiation has not yet occurred [13].

The combination of limb deformities and congenital heart defects necessitates differentiation from other syndromes with overlapping phenotypes. Thrombocytopenia‐absent radius (TAR) syndrome, characterized by radial aplasia, is a potential differential diagnosis. However, unlike HOS, TAR syndrome is associated with thrombocytopenia and spares the thumb [14]. Another differential diagnosis is the VACTERL association, which includes vertebral, anal, cardiac, tracheoesophageal, renal, and limb anomalies, but it lacks the genetic basis of HOS [15]. Lastly, Fanconi anemia is a syndrome that presents with skeletal anomalies and cardiac defects but is also characterized by bone marrow failure and an increased risk of malignancy [16]. The presence of bilateral skeletal abnormalities, congenital heart disease, and the absence of hematologic abnormalities strongly support the diagnosis of HOS over these conditions.

Although molecular confirmation of TBX5 mutations is the definitive diagnostic method [2], it is not widely available in Ethiopia. As a result, diagnosis relies primarily on clinical criteria, which include upper limb anomalies affecting the radial ray (e.g., thumb hypoplasia, carpal fusion), cardiac defects, typically affecting septal structures, and a positive family history (although de novo mutations are common) [17]. The absence of genetic testing in our setting underscores the need for increased clinical awareness to diagnose HOS based on phenotypic features alone. The use of echocardiography and limb radiographs remains essential for proper diagnosis in low‐resource environments.

To confirm that this is the first reported case of HOS from Ethiopia, we conducted a comprehensive literature search using PubMed, Scopus, Elsevier, and EBSCOhost databases. The search was performed using the following keywords and Boolean operators: (“Holt‐Oram syndrome” OR “heart‐hand syndrome” OR “TBX5 mutation”) AND (“Ethiopia” OR “Ethiopian” OR “East Africa” OR “sub‐Saharan Africa”). Additionally, we searched the African Journals Online (AJOL) database to identify any regional case reports. The search was limited to articles published in English with no date restrictions. This search yielded no published case reports of HOS from Ethiopia or the East African region, supporting our assertion that this represents the first documented case from Ethiopia.

What makes this case unique compared with typical congenital heart disease cases? First, unlike isolated congenital heart disease, this case demonstrates the classic HOS triad of cardiac defects (membranous VSD with severe pulmonary hypertension), upper limb anomalies (absent thumb, radial hypoplasia, carpal fusion), and characteristic skeletal deformities. Second, although VSD is a common cardiac defect, the presence of severe pulmonary hypertension (RVSP > 50 mmHg) with moderate‐to‐severe mitral regurgitation and severe tricuspid regurgitation at age 18 represents an advanced disease state rarely documented in HOS literature. Third, this case highlights the diagnostic challenges and successful clinical recognition of a rare genetic syndrome in a setting where genetic testing is unavailable, relying solely on clinical criteria. Fourth, as confirmed by our literature search, this is the first documented case of HOS from Ethiopia and one of few from sub‐Saharan Africa.

Cardiac defects in HOS impact morbidity and mortality. Management aims to optimize cardiac function with diuretics, monitor for pulmonary hypertension and conduction abnormalities, and consider surgical repair when needed. Early VSD correction is ideal, but because of the limited surgical access, symptomatic treatment and follow‐up are emphasized [18]. Management also includes physical therapy for upper limbs and pharmacological treatments for heart failure and arrhythmias in symptomatic patients [19].

## 4. Conclusion

This is the first case report of HOS from Ethiopia. This assertion is supported by a comprehensive literature search of PubMed, Scopus, Elsevier, EBSCOhost, and AJOL databases, which yielded no previously published cases of HOS from Ethiopia or the East African region. The diagnosis needs meticulous physical assessment, imaging techniques, and a thorough familial medical history. This case elucidates the hallmark features observed in a young Ethiopian male patient presenting with upper limb and cardiac defects. It underscores the possibility of diagnosing rare genetic anomalies such as HOS using the clinical criteria, despite the genetic analysis being neither accessible nor affordable in a resource‐limited environment.

## Author Contributions

Y.G. conceived the study. A.B., S.M., B.A., and B.B. researched the literature review and prepared the manuscript. A.M., B.D., A.Y., E.F., and B.F. diagnosed the patient and drafted the initial manuscript. E.F., I.K., and B.F. wrote the presentation of the case and contributed to the discussion section. B.A. edited and coordinated the manuscript.

## Funding

No funding was received for this manuscript.

## Disclosure

All authors have read and approved the final manuscript.

## Ethics Statement

Our institution does not require ethical approval for reporting individual cases or case series.

## Consent

Written informed consent was obtained from the patient for publication of this case report and any accompanying images. A copy of the written consent is available for review by the editor in chief of this journal.

## Conflicts of Interest

The authors declare no conflicts of interest.

## Data Availability

Data sharing is not applicable to this article as no datasets were generated or analyzed during the current study.
